# Glandular urethral disassembly for Effmann type I A1 urethral duplication with glans duplication: a case report

**DOI:** 10.3389/fped.2026.1815533

**Published:** 2026-04-29

**Authors:** Rafael Enrique Jordan Balladares, Oscar Caloca Ibarra, Fernando Aguilar Torres, David Vazquez Perez, Barbara Rivera Pereira, Sergio Landa Juarez

**Affiliations:** Hospital de Especialidades, Centro Medico Nacional Siglo XXI, Instituto Mexicano del Seguro Social, Mexico City, Mexico

**Keywords:** case report, Effman classification, glandular urethral disassembly, glans duplication, pediatric urology, urethral duplication

## Abstract

**Background:**

Urethral duplication is a rare congenital anomaly with heterogeneous anatomical presentations. Effmann type I A1 represents an incomplete distal duplication and is rarely associated with glans duplication.

**Case presentation:**

We report the case of a 10-year-old boy presenting with a bifid glans and double distal meatus causing urinary spraying. Imaging and endoscopic evaluation confirmed Effmann type I A1 urethral duplication with associated glans duplication. Surgical reconstruction was performed using glandular urethral disassembly (GUD) with excision of the accessory urethra and glans reconstruction.

**Results:**

The postoperative evolution was uneventful. At the 6-month follow-up, the patient demonstrated a single urinary stream, normal voiding, no fistula or stenosis, and a satisfactory cosmetic outcome.

**Conclusion:**

This report describes the first application of GUD for Effmann type I A1 urethral duplication with glans duplication. This technique is a feasible and effective reconstructive option for selected distal urethral duplications.

## Introduction

Urethral duplication is a rare congenital anomaly with an estimated incidence of fewer than 1 in 1,000,000 live births (1, 2). Since its initial description, fewer than 350–500 cases have been documented worldwide (1, 3), predominantly in male patients (4). The Effmann classification categorizes urethral duplication (4). Type I A1 corresponds to an incomplete distal duplication, defined as a complete or incomplete accessory urethra that does not communicate with the primary urethral tract or the bladder.

The complexity of this case was further compounded by its association with glandular diphallia (partial penile duplication). Association with glans duplication is exceedingly rare, with only isolated case descriptions available. Diphallia itself is a rare malformation, occurring in approximately 1 per 5–6 million births (5, 6), while glandular diphallia is a localized failure in the fusion of the distal genital tubercle, according to the classification by Jesus et al. (7). The synchronous presentation of an Effmann type I A1 duplication within a bifid or double glans is a clinical “rarity within a rarity,” posing significant challenges for both functional preservation and aesthetic reconstruction.

Glandular urethral disassembly (GUD), originally described by Dr. Antonio Macedo et al. (8) for the repair of distal hypospadias, allows complete mobilization of the glandular urethra while preserving vascular supply. Its application in urethral duplication has not been reported. We present a novel use of GUD for the management of Effmann type I A1 urethral duplication associated with glans duplication.

## Case presentation

A 10-year-old boy was referred to our pediatric urology clinic for evaluation of a suspected penile malformation. Family medical history was unremarkable, with no known history of urinary or genital malformation among relatives. There was no past medical history of chronic illness, previous hospitalizations, or surgical procedures. The patient had no history of urinary tract infections or voiding dysfunction. His main complaint was an abnormal urinary stream direction, with the urine spraying downward, which frequently caused urine to spill outside the toilet. Because of this, the patient avoided using public restrooms and changing rooms with peers, expressing reluctance for other children to see his genitalia.

**Figure 1:**
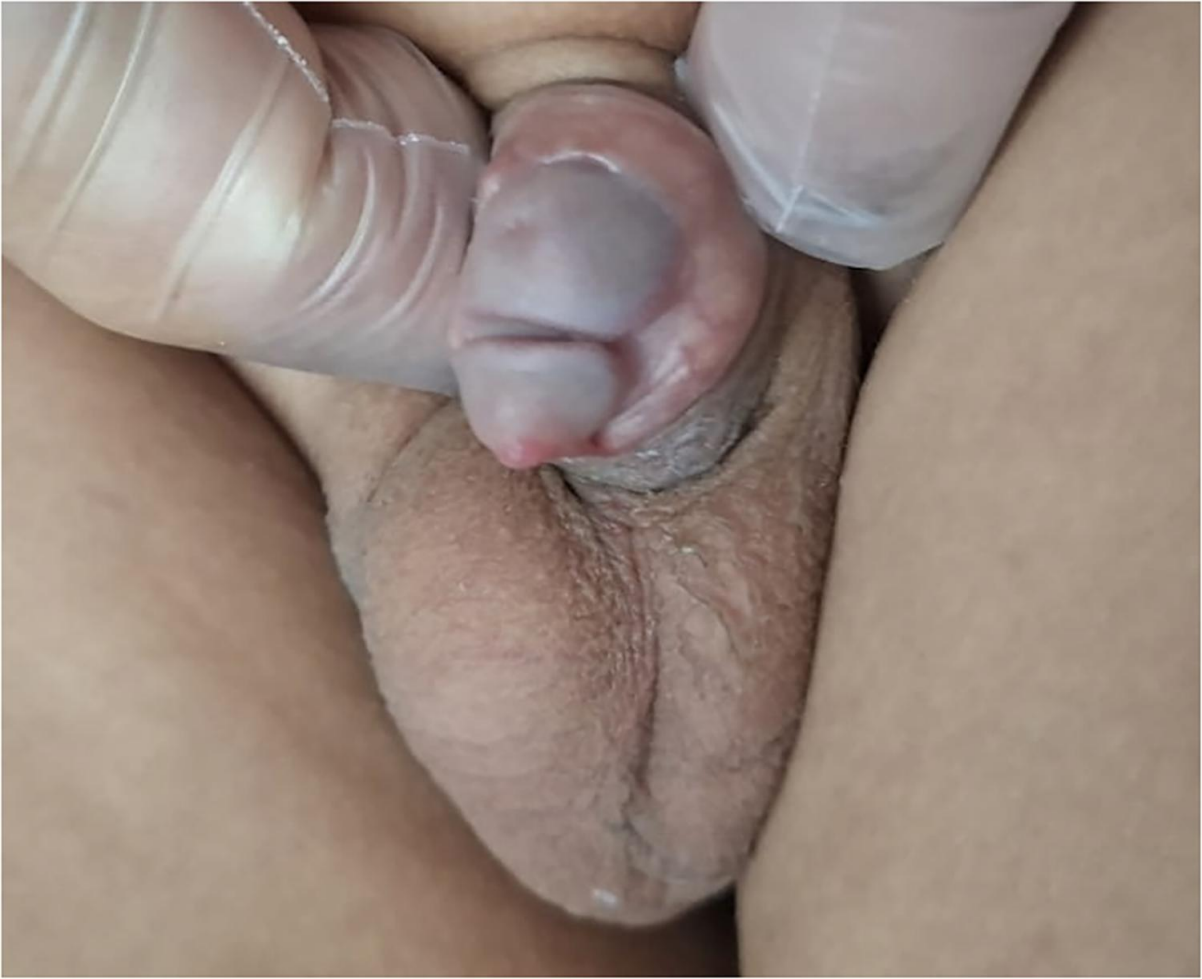
Preoperative view showing partial glans duplication (bifid glans) with two distal meatal openings located at the tip pf the glans.

On physical examination, the penis was normally developed in length and shaft morphology, with no chordee or penile curvature. The glans demonstrated a partial duplication (bifid configuration), with two distal openings located at the tip of the glans separated by a shallow median cleft (Figure 1). Both meatuses were located distally within the glandular tissue and appeared independent. The surrounding spongiosal tissue was palpable and appeared symmetrical, without palpable fibrosis or abnormal masses. The penile shaft was otherwise normal, with no evidence of hypospadias, epispadias, or skin abnormalities. The scrotum was normally formed, with bilaterally descended testes. No other external genital anomalies were identified.

Given the unusual glandular configuration and presence of two distal meatal openings, endoscopic evaluation was performed to accurately characterize the urethral anatomy and determine whether the duplication represented a complete or incomplete variant.

Cystoscopy was performed, demonstrating a single normal orthotopic urethra coursing through the anterior glandular segment, with normal caliber and mucosa and an incomplete duplicated urethral channel originating at the posterior aspect of the glans. This confirmed distal incomplete urethral duplication consistent with Effmann type I A1.

## Surgical technique

Surgery was performed under combined anesthesia. A non-absorbable traction suture was placed at the glans to facilitate exposure. An 8 Fr catheter was inserted into the urethra to identify and protect the main urethral lumen.

Complete penile degloving was performed, preserving a small ventral skin bridge distal to the meatus to facilitate urethral manipulation. The duplicated glans components were carefully separated. The urethra was carefully mobilized and the duplicated accessory urethral channel was dissected proximally within the glans and excised completely.

**Figure 2:**
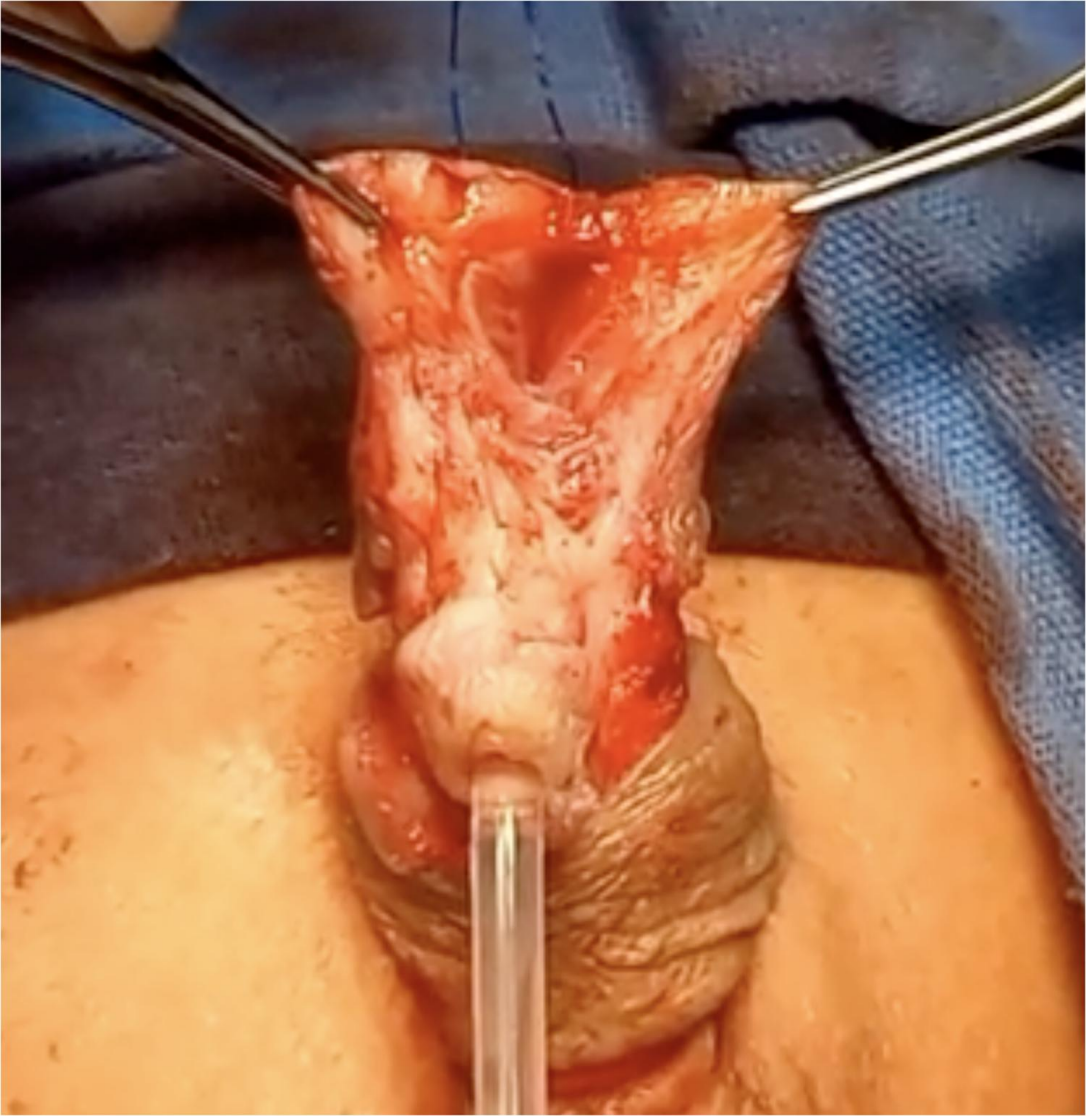
Intraoperative view demostrating glandular urethral disassembly (GUD). The glans has been separated into two hemi-glandular components, allowing identification of the native urethra and dissection of the accessory urethral channel.

**Figure 3:**
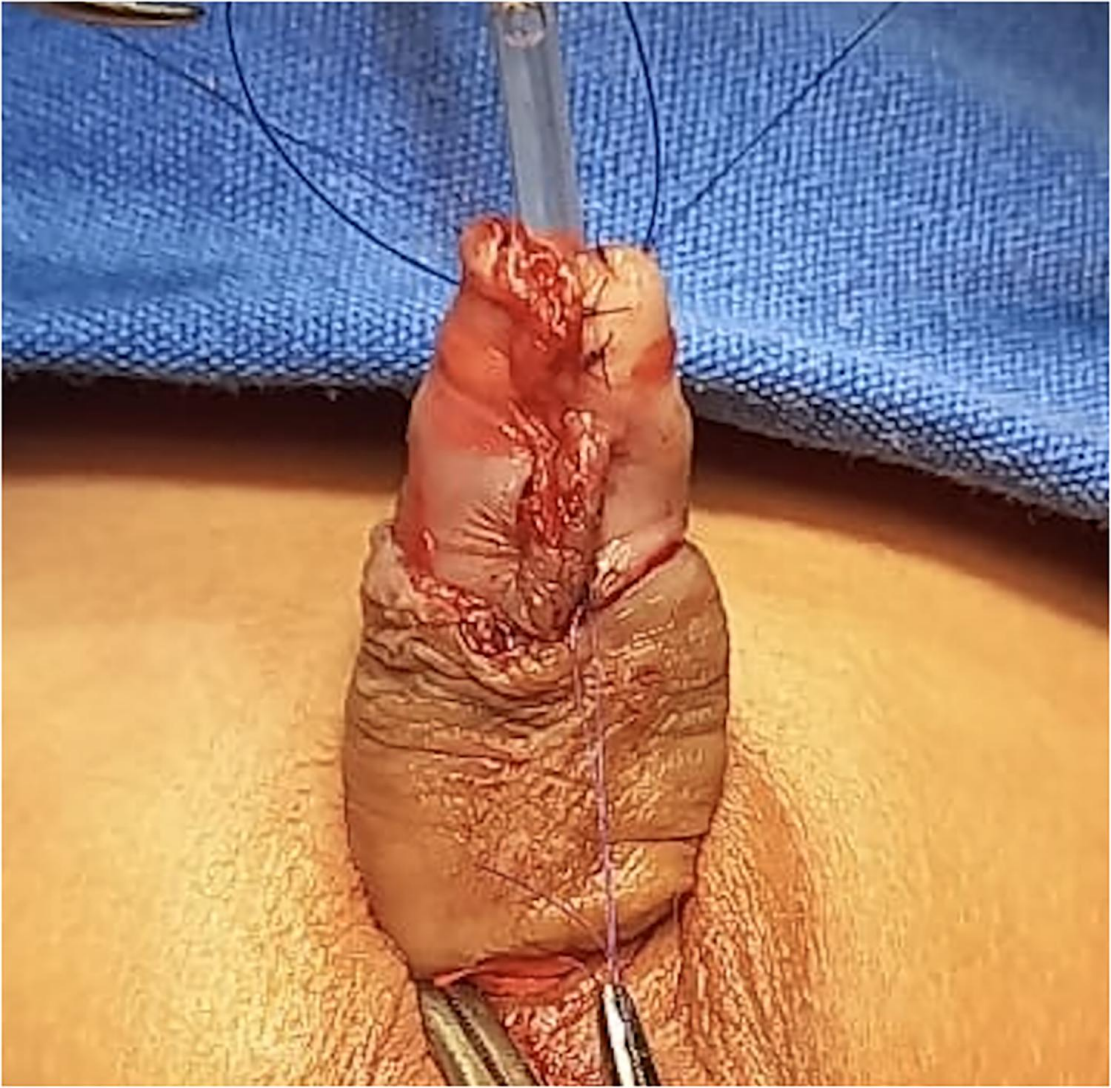
Final intraoperative appearance after excision of the accessory urethra and glandular reconstruction, showing a single orthotopic meatus whith restored conical glans configuration.

The urethra was repositioned centrally within the glans. The glans was opened into two wide hemi-glandular wings using an inverted “Y” incision (Figure 2). The native urethra was secured to the glans with absorbable sutures (6-0) at the 11, 2, 7, and 4 o'clock positions to ensure stable centralization. A spongioplasty was performed to provide additional soft tissue coverage and support for the reconstructed urethra. The glans was then closed over the urethra in layers, recreating a conical glans configuration with a single orthotopic meatus (Figure 3).

The Foley catheter was secured and left in place for approximately 10 days postoperatively.

## Results

The postoperative evolution was uneventful. The catheter was removed on postoperative day 10. At the 1-, 3-, and 6-month follow-ups, the patient reported a single urinary stream without spraying or dysuria. The glans and meatus had a satisfactory cosmetic appearance.

## Discussion

Urethral duplication is a rare congenital anomaly characterized by a wide variety of anatomical alterations. The classification proposed by Effmann et al. remains the most widely used system for categorizing these anomalies. Type I A1 corresponds to an incomplete distal duplication, in which the accessory urethra does not communicate with either the bladder or the native urethra (4).

The epidemiology and clinical presentation of urethral duplication have been described by numerous authors. Salle et al. reviewed 16 cases and highlighted the marked anatomical heterogeneity of this condition, as well as the need to individualize treatment according to the anatomical configuration and symptoms (1). Similarly, Podesta et al. and Mane et al. published pediatric series demonstrating that the surgical approach must be adapted to the specific anatomical subtype and clinical manifestations (2, 3).

Recent evidence further supports these observations. A systematic review by Gozar et al. identified 250 patients across 90 studies, confirming the extreme rarity of urethral duplication; the majority of the studies were case reports or had a small series of patients (9). This systematic review highlights the significant diversity in anatomical presentations and the lack of standardized management guidelines. Classification systems such as the Effmann classification help categorize anatomical variables, but they do not determine surgical management, which remains highly individualized and depends on the specific anatomical features and the surgeon's experience.

Incomplete distal duplications, such as Effmann type I A1, are the least complex variants. In the majority of patients, this condition may be asymptomatic and even discovered incidentally (2). However, distal duplication associated with glans anomalies, being a more complex variation, can produce functional symptoms such as urinary spraying, abnormal stream direction, or aesthetic problems that warrant surgical correction.

An association between urethral duplication and glans duplication is rare. Glans duplication (or diphallia) is an extremely rare congenital anomaly (1 in 5 million) where the penis presents with two glandes, or in more severe cases, two complete penises, resulting from incomplete development or fusion of the distal genital tubercle during embryogenesis (6).

Several classifications of glans duplication have been proposed. Jesus et al. analyzed this anomaly and suggested that glandular duplication can occur as an isolated finding or in association with urethral anomalies (7). Similarly, Aihole et al. reported a case of glans duplication associated with urethral duplication, highlighting the reconstructive challenges posed by this anatomical combination (5).

Surgical management depends primarily on the anatomical subtype and clinical presentation. In incomplete distal duplications, excision of the accessory urethra is commonly considered an appropriate treatment when symptoms are present (1–3). However, when the duplication involves the glans, surgical planning must address not only the removal of the accessory duct but also the reconstruction of the glandular anatomy, preserving vascular integrity.

In this case, the anatomical configuration required both excision of the accessory urethra and reconstruction of a split glans. For this reason, we opted to use the GUD technique described by Macedo et al. for distal hypospadias repair (8). This technique allows for complete mobilization of the glandular urethra, preserving its vascular supply, which facilitates glans reconstruction and adequate mobilization of the native urethra.

The structural principles of the GUD technique make it adaptable to other distal urethral anomalies requiring glandular reconstruction. In our case, the technique allowed for clear identification of the native urethra, safe resection of the accessory urethral canal, and the configuration of a tension-free glans, leaving a single meatus at the tip. This case expands the application of GUD beyond hypospadias and suggests its usefulness in selected cases of distal urethral duplication with glandular involvement.

This report is limited by being a single case, reflecting the rarity of the condition, and by the relatively short follow-up period of 6 months. Despite these limitations, this case contributes to the existing literature by documenting a rare anatomical association and demonstrating the feasibility of adapting the GUD technique for distal urethral duplication reconstructions with glans involvement.

## Conclusion

GUD is a safe and effective reconstructive option for Effmann type 1 A1 urethral duplication associated with glans duplication. This novel application expands the surgical armamentarium for rare distal urethral anomalies.

## Data Availability

The raw data supporting the conclusions of this article will be made available by the authors, without undue reservation.
